# Integrating Management Practices to Decrease Deoxynivalenol Contamination in Soft Red Winter Wheat

**DOI:** 10.3389/fpls.2020.01158

**Published:** 2020-07-30

**Authors:** Katherine S. Rod, Carl A. Bradley, David A. Van Sanford, Carrie A. Knott

**Affiliations:** ^1^ Department of Plant and Soil Science, University of Kentucky, Lexington, KY, United States; ^2^ Department of Plant Pathology, University of Kentucky, Princeton, KY, United States; ^3^ Department of Plant and Soil Science, University of Kentucky, Princeton, KY, United States

**Keywords:** Fusarium head blight, FHB, anthesis uniformity, in-furrow phosphorus, seeding rate, harvest timing

## Abstract

*Fusarium graminearum*, the major causal agent of Fusarium head blight (FHB) of wheat (*Triticum aestivum*) in the U.S., can produce mycotoxins, such as deoxynivalenol (DON), during infection. Contamination of wheat grain with DON is a major concern for wheat producers and millers, and the U.S. Food and Drug Administration (FDA) has set advisory levels for DON in finished wheat products for human and animal consumption. Practices utilized to manage FHB and DON contamination include planting wheat cultivars with moderate resistance to FHB and applying efficacious fungicides at the beginning of anthesis. Under severe epidemics, DON contamination can exceed FDA advisory levels despite implementation of these measures. Additionally, fungicide efficacy can be limited when anthesis is not uniform among plants in the field, which can occur when planting is delayed or if there is non-uniform seedling establishment. The objectives of this study were to evaluate the effect of (1) in-furrow phosphorus application at planting and seeding rate on heading and anthesis uniformity, FHB symptomology, DON contamination, grain yield, yield components, and test weight; and (2) harvesting at different grain moisture concentrations on FHB symptomology, DON contamination, grain yield and test weight. Field trials were established in Princeton, Kentucky, from 2017 to 2019, to evaluate in-furrow phosphorus application at planting (0 kg P_2_O_5_ ha^-1^ and 47 kg P_2_O_5_ ha^-1^); seeding rate (377 live seeds m^-2^ and 603 live seeds m^-2^); and grain moisture at harvest (20 to 22% and 13 to 15%). In-furrow phosphorus increased grain yield and spikes m^-2^, but had no effect on heading and anthesis uniformity or DON contamination. The 603 live seeds m^-2^ seeding rate decreased the number of days to Zadoks 60 for the November planted wheat, and decreased FHB incidence, but did not decrease DON contamination. Harvesting at 20 to 22% grain moisture decreased Fusarium damaged kernel ratings and percent kernel infection but increased DON contamination in the harvested grain. Although in-furrow phosphorus, seeding rate, and harvesting 20 to 22% grain moisture did not decrease DON contamination, there is potential for these treatments to alleviate negative effects of late planted wheat grown in stressful environments.

## Introduction

*Fusarium graminearum* (Schwabe) is the major cause of Fusarium head blight (FHB) in wheat (*Triticum aestivum* L.) in the United States and can produce mycotoxins, such as deoxynivalenol (DON) during infection (Andersen, 1948). Fusarium head blight is usually a regional problem with severe infection occurring in years where environmental conditions are warm (25°C) and moist during anthesis through soft dough stage, furthermore, severe epidemics can occur when conditions are favorable for infection, which can lead to reduced grain yield (Andersen, 1948; McMullen et al., 2012). However, the major concern to wheat producers and millers is DON contamination, as the mycotoxin can cause adverse health effects on humans and animals if consumed in high levels. The U.S. Food and Drug Administration has set advisory levels for DON in finished wheat products for humans at 1 ppm, swine at 5 ppm, and cattle at 10 ppm (FDA, 2010). Current management practices include planting moderately resistant cultivars and applying efficacious fungicides at beginning anthesis to control FHB and DON contamination (Zadoks 60) (Wegulo et al., 2011; Willyerd et al., 2012; Wegulo et al., 2015). Integrating cultivar resistance and fungicide application at anthesis can be an effective practice to manage FHB and DON contamination (Wegulo et al., 2011; Willyerd et al., 2012). Despite implementing these management practices, when environmental conditions are favorable for *F. graminearum* infection, DON contamination can exceed FDA advisory levels (McMullen et al., 2012). Additional management practices are needed to mitigate the limitations of current recommendations as DON contamination can sometimes reach critical levels even when FHB symptoms are not severe (Cowger and Arrellano, 2010; Knott, 2014; Andersen et al., 2015).

Additional agronomic management practices may need to be implemented to better mitigate FHB and DON contamination. For example, in-furrow phosphorus and seeding rates can alter tiller development, potentially creating a more uniform head development and anthesis (Schaafsma and Tamburic-Ilincic, 2005; Otteson et al., 2008; Chen et al., 2019). Several tillers on a wheat plant can develop fertile spikes and shed pollen across a range of days as all of these tillers are not at the same development stage during heading and anthesis (Kiesselbach and Sprague, 1926; Noversoke, 2014; Tilley et al., 2019). More uniform spike development could lead to more uniform anthesis and better fungicide efficacy, as current fungicides (prothioconazole + tebuconazole and metconaxole) have been shown to reduce FHB incidence by only approximately 50% compared to a non-treated control (Paul et al., 2018). Phosphorus can be applied in a variety of placements in the soil during fall application; however in-furrow application at planting has been shown to result in greater grain yield compared to broadcast application (Peterson et al., 1981; Fiedler et al., 1989; Grant et al., 2001). Phosphorus has also been shown to increase early-season tiller development, which has resulted in increased spikes m^-2^ and grain yield (Knapp and Knapp, 1978; Blue et al., 1990; Sander and Eghball, 1999; Chen et al., 2019). Knapp and Knapp (1978) observed that wheat fertilized with fall applied phosphorus reached Zadoks 58 (100% of spike visible) earlier than wheat planted without phosphorus. It is speculated that plants with vigorous early season tiller development may have more of the plant’s tillers at the same growth stage at spike development and anthesis. Another management practice that may alter tiller development is increased seeding rate. Increasing the seeding rate can increase the number of main stem and primary tillers m^-2^ (Blue et al., 1990; Geleta et al., 2002; Lloveras et al., 2004) while decreasing the number of secondary and tertiary tillers per unit area (Kiesselbach and Sprague, 1926; Lloveras et al., 2004; Otteson et al., 2008; Tilley et al., 2019). Decreasing the number of non-main stem tillers does not usually decrease yield, as the main spike and primary tiller are the major contributors to grain yield (Otteson et al., 2008; Chen et al., 2019). In theory, having more main stems and primary tillers per unit area would result in more spikes that are uniform in their developmental stage, setting up a scenario which could lead to greater fungicide efficacy.

Another management practice that may decrease DON contamination in harvested wheat grain is harvest timing. Anecdotal observations from producers suggest that harvesting wheat at a grain moisture greater than 15% could decrease DON contamination in the harvested grain compared to harvesting grain at a moisture less than 15%. Current harvest strategies to decrease DON contamination in harvested grain include optimizing combine harvester settings and air speed to remove Fusarium damaged kernels (FDK) from entering the grain tank (Salgado et al., 2011). However, wheat harvested in Ontario, Canada, at approximately 18 to 25% grain moisture has been shown to have a lower incidence of *Fusarium* spp. kernel infection leading to higher grain quality, although there was no difference in DON contamination compared to grain harvested at less than 15% grain moisture (Xue et al., 2004). Similarly harvesting corn (*Zea mays* L.), when grain moistures are greater than 15% has been shown to reduce the amount of mycotoxin, as continual mycotoxin development can occur as the corn dries slowly in the field, compared to harvesting at less than 15% grain moisture (Munkvold, 2003). Even though *F. graminearum* and DON can continue to accumulate when wheat grain moisture is greater than 17% (Hope et al., 2005), potentially harvesting at greater than 15% grain moisture could lower mycotoxin levels in soft red winter wheat.

The goal of this study was to determine whether additional management practices would increase anthesis uniformity, decrease DON contamination, and improve grain yield and quality. The specific objectives of this study were to evaluate the effect of: (1) in-furrow phosphorus application at planting and seeding rate on heading and anthesis uniformity, FHB symptomology [including FHB incidence, FHB severity, FHB index, Fusarium damage kernel (FDK) rating, and percent kernel infection (PKI)], DON contamination, grain yield, yield components, and test weight; and (2) harvesting at different grain moisture concentrations on FHB symptomology, DON contamination, grain yield, thousand kernel weights (TKW) and test weight.

## Materials and Methods

### Environments and Experimental Design

Soft red winter wheat trials were established at the University of Kentucky Grain and Forage Center of Excellence at the Research and Education Center in Princeton KY (37°6’ N, 87°52’ W) in the fall of 2016, 2017, and 2018. In each year, a total of four environments were established. Three environments were established on a Crider silt loam (fine-silty, mixed, active, mesic Typic Paledalf) and one was established on a Zanesville silt loam (fine-silty, mixed, active, mesic Oxyaquic Fragidalf) (**Table 1**). Plots were planted with a research no-till drill (Plotseed XL; Wintersteiger Inc., Salt Lake City, UT) into corn stubble. Rows were spaced 18 cm apart and each plot was approximately 1.2 m wide and 4.6 m long. Two of the Crider environments were infested with *F. graminearum* inoculated corn kernels and mist irrigated, creating high FHB disease pressure conditions (Verges et al., 2006). The remaining Crider environment and Zanesville environment were not infested with *F. graminearum* inoculated corn kernels or mist-irrigated. Each environment was arranged as a split-split plot randomized complete block design. The main plot was harvest timing, which consisted of two target harvest moistures: a high moisture harvest at 20 to 22% grain moisture (early) and a normal moisture harvest at 13 to 15% grain moisture (normal). The split-plot was planting timing, which consisted of two planting timings of October and November (**Table 1**). Throughout the rest of the article, the harvest timing and planting timing combinations will be referred to as: October early harvest, October normal harvest, November early harvest, and November normal harvest. In 2016, the split-split plot consisted of four management treatments, replicated five times: two in-furrow phosphorus rates (0 kg ha^-1^ P_2_O_5_ or 47 kg ha^-1^ P_2_O_5_), and two cultivars (moderately resistant to FHB cultivar [Pembroke 2016; Kentucky Small Grain Growers Association, Eastwood, KY] or moderately susceptible to FHB cultivar [Cumberland; Kentucky Small Grain Growers Association, Eastwood, KY]). In 2017 and 2018, the split-split plot consisted of eight management treatments, replicated five times: two in-furrow phosphorus rates (0 kg ha^-1^ P_2_O_5_ or 47 kg ha^-1^ P_2_O_5_), two seeding rates (377 plants m^-2^ or 603 plants m^-2^), and two cultivars (moderately resistant to FHB cultivar [Pembroke 2016; Kentucky Small Grain Growers Association, Eastwood, KY] or moderately susceptible to FHB cultivar [Pioneer 26R53; Corteva, Johnston, IA]). Wheat plots were managed for nitrogen and herbicide applications according to University of Kentucky Cooperative Extension Service recommendations (Lee et al., 2009).

**Table 1:** Environmental conditions of soil type, *Fusarium graminearum* infestation type, fungicide application, planting dates, and harvest dates of soft red winter wheat trials in Princeton, Kentucky, USA from 2017 to 2019.

Environment	Soil Type	*F.* *graminearum* Infestation Type	Fungicide Application at Zadoks 60	Planting Date			Harvest Date		
				2016	2017	2018	2017	2018	2019
**1**	Crider Silt Loam	Infested with *F. graminearum* inoculated scabby corn kernels	No	25-Oct	18-Oct	11-Oct	08-Jun	08-Jun	12-Jun
							21-Jun	19-Jun	13-Jun
				22-Nov	28-Nov	28-Nov	12-Jun	15-Jun	28-Jun
							21-Jun	02-Jul	28-Jun
**2**	Crider Silt Loam	Infested with *F. graminearum* inoculated with scabby corn kernels	Yes^a^	25-Oct	18-Oct	11-Oct	08-Jun	08-Jun	12-Jun
							21-Jun	02-Jul	13-Jun
				22-Nov	28-Nov	28-Nov	12-Jun	15-Jun	28-Jun
							21-Jun	02-Jul	28-Jun
**3**	Crider Silt Loam	No *F. graminearum* inoculum was applied	Yes	15-Oct	18-Oct	09-Oct	08-Jun	08-Jun	11-Jun
							21-Jun	02-Jul	13-Jun
				22-Nov	28-Nov	28-Nov	12-Jun	15-Jun	28-Jun
							21-Jun	02-Jul	28-Jun
**4**	Zanesville Silt Loam	No *F. graminearum* inoculum was applied	Yes	15-Oct	19-Oct	11-Oct	–	08-Jun	12-Jun
							–	02-Jul	13-Jun
				22-Nov	28-Nov	28-Nov	–	15-Jun	28-Jun
							–	02-Jul	–

Each environment had two planting timings (October and November) and each planting timing had two harvest timings (targeted at 20 to 22% grain moisture and 13 to 15% grain moisture).

^a^In 2017 and 2018, Prothiconazole + Tebuconazole (Prosaro, Bayer Crop Science, St. Louis, MO) were applied at a rate of 0.1 kg a.i. ha^-1^ + 0.1 kg a.i. ha^-1^ and 0.0125% v/v non-ionic surfactant (Ad-Spray 80, Helena Chemical Company, Collierville, TN) was added to the spray solution. In 2019, Caramba (Metconazole, BASF, Research Triangle Park, NC) was applied at a rate of 0.1 kg a.i. ha^-1^ + and 0.125% v/v non-ionic surfactant was added to the spray solution.

Fungicide applications for FHB were applied to three of the four environments each year (**Table 1**) when wheat was at Zadoks 60 (beginning anthesis) growth stage. In 2017 and 2018, prothioconazole + tebuconazole (Prosaro, Bayer CropScience, St. Louis, MO) was applied at 0.1 kg a.i. ha^-1^ + 0.1 kg a.i ha^-1^ and 0.0125% v/v non-ionic surfactant (Ad-Spray 80, Helena Chemical Company, Collierville, TN) was included in the spray solution. In 2019, the fungicide metconazole (Caramba, BASF Corp., Research Triangle Park, NC) was applied at a rate of 0.1 kg a.i. ha^-1^ and 0.125% v/v non-ionic surfactant was added to the spray solution.

### Wheat Heading and Anthesis Uniformity

Wheat heading and flowering uniformity were measured in the spring of 2018 and 2019 in the Crider ambient environment. In 2018, once a day, for approximately two weeks for each planting timing, approximately 0.6 m of one row of wheat heads per plot was photographed with a Canon Power Shot camera (Elph 115IS, Canon, Melville, NY). The pictures were analyzed for the number of wheat heads that were at growth stage Zadoks 58, Zadoks 60, and Zadoks 68 each day. In 2019, growth stages (Zadoks 58, Zadoks 60, and Zadoks 68) were measured in the field for approximately 2 weeks for each planting timing.

### Fusarium Head Blight Symptomology 

One meter row of spikes were hand harvested per plot approximately 17 to 20 days after Zadoks 68. These spikes were placed in a walk in freezer (-19°C, Hobart, Troy, OH) to preserve the samples until spikes m^-2^, number of spikelets spike^-1^, FHB incidence, FHB severity, and FHB index could be determined. Spikes m^-2^ and FHB incidence (% of plants with FHB symptoms) were measured from the entire sample. Number of spikelets spike^-1^ were measured from 15 random spikes, and FHB severity (% head area affected) was measured from 15 FHB symptomatic spikes. Fusarium head blight index were determined using the equation FHB index=(FHB incidence x FHB severity)/100.

### Wheat Harvest

Wheat was harvested using a small plot combine (Delta; Wintersteiger, Inc., Salt Lake City, UT) equipped with a weigh system (Harvest Master, Juniper Systems, Inc., Logan, UT). Approximately 1.4 kg of grain were collected from each plot. The wheat harvested at the target high grain moisture of 20 to 22% was stored in plastic bags in a walk in cooler (4°C, Forma-Kool, Chesterfield, MI) until the wheat could be dried to 12.5% grain moisture with a laboratory-scale thin-layer drying system (White et al., 1985). Wheat harvested at target normal grain moisture of 13 to 15% was stored in paper bags at 22°C and 50% relative humidity. Despite enduring harvest challenges, some years the target harvest moisture of 20 to 22% was unable to be met, however grain was harvested as early as possible (**Table 2**). The Zanesville 2017 environment was unable to be harvested due to environmental factors, and the Zanesville 2019 November normal harvest was unable to be harvested due to poor plant emergence and end season plant density (**Table 2**). Grain moisture and test weight were measured using a Dickey-John Grain Analysis Computer (GAC) (Model 2500-UGMA; Dickey-John, Auburn, IL) immediately after harvest and drying. Test weight was only measured in 2018 and 2019. Grain yields were adjusted to 13.5% grain moisture.

**Table 2:** Target and actual harvest grain moistures for all environments from soft red winter wheat trials from Princeton KY 2017 to 2019.

Environment	Planting Timing	Target Grain Moisture (%)	Harvest 2017	Harvest 2018	Harvest 2019
			Actual Grain Moisture (%)	Actual Grain Moisture (%)	Actual Grain Moisture (%)
**1**	October	20 to 22	11.9	17.3	15.5
		13 to 15	13.8	15.0	15.5
	November	20 to 22	14.4	19.0	14.3
		13 to 15	14.8	15.0	13.3
**2**	October	20 to 22	12.4	20.9	15.7
		13 to 15	13.8	13.9	15.5
	November	20 to 22	15.5	20.0	13.8
		13 to 15	14.5	12.9	12.9
**3**	October	20 to 22	16.4	25.1	21.2
		13 to 15	15.5	12.3	18.9
	November	20 to 22	15.5	23.2	15.0
		13 to 15	14.8	11.7	12.4
**4**	October	20 to 22	–^a^	17.8	15.6
		13 to 15	–	12.5	14.7
	November	20 to 22	–	25.1	16.1
		13 to 15	–	13.8	–^b^

a Zanesville 2017 (Environment 4) was not able to be harvested due to environmental conditions.

b The Zanesville 2019 (Environment 4) November normal harvest was not able to be harvested due to poor stand emergence and poor end season plant density.

Thousand kernel weights (TKW), FDK ratings, and DON contamination were measured post-harvest. One thousand kernels were counted with a Seedburo Seed Counter (Model 801; Des Plaines, IL) and then weighed to determine TKW. Thousand kernel weights were then adjusted to 13.5% grain moisture. A random grain sample of approximately 200g was visually inspected for percentage of Fusarium damaged kernels based on a visual scale similar to Paul (2015). This was repeated twice, for a total of three observations, per plot and then averaged to determine FDK rating for each plot. A random 100g sample from each plot was ground into a rough wheat meal powder using a Romer Series II mill (Romer Labs, Inc., Union MO). The mill was vacuumed cleaned between each sample. A 20g subsample was used for the DON analysis per the protocol for QuickTox for QuickScan DON Flex kits by Envirologix (Portland, ME). Analyses were preformed using the provided protocol and DON contamination levels were determined using the QuickScan system (Envrionlogix, Portland, ME). A subsample of grain from a select number of plots each year was also sent to the University of Minnesota to validate DON contamination using gas-chromatography, mass-spectrometry.

### Percent Kernel Infection

Twenty asymptomatic kernels from each plot were surface disinfested by soaking in a 10% bleach water solution for 1 min, then a sterile water rinse, followed by soaking in 90% ethanol for 1 min. Kernels were then triple rinsed in sterile water before aseptically transferred to peptone pentacholoronitrobenze (PCNB) agar Dill-Macky (2003). This was repeated four times for a total of five runs. Plates were stored in a growth chamber (Model: I-36VL, Percival, Perry IA), programed to run 12 h at 24.7°C with light and 12 h at 22°C without light. Percent kernel infection (PKI) was measured six days after plating; infected kernels were defined as kernels with visual signs of *Fusarium* spp. pink mycelium. *Fusarium* spp. was not identified to specific species.

### Data Analyses 

Normality was confirmed (PROC UNIVARIATE; SAS, v9.4; SAS Institute Inc., Cary, NC) prior to analyses of variance (PROC GLIMMIX; SAS, v9.4) for days to beginning anthesis (Zadoks 60), spikes m^-2^, spikelets spike^-1^, TKW, PKI, grain yield, and test weight. To obtain normally distributed data, FDK ratings were arcsine square root transformed, while DON contamination, FHB Incidence, FHB severity, and FHB index were log transformed.

Analyses of variance were initially performed (PROC GLIMMIX; SAS, v9.4) for a full model that included year, environment, main effect, and all possible combinations (specified in SAS as year|environment|main effect) as fixed effects and replication as random effects. Significant (*P* < 0.05) interactions did not exist between year and any of the main effects or environment and any of the main effects for all dependent variables. In addition, significant (*P* < 0.05) interactions were not detected for any of the dependent variables (listed above) for in-furrow phosphorus by seeding rate, in-furrow phosphorus by cultivar, or seeding rate by cultivar. There was a significant (*P* < 0.05) planting timing by seeding rate interaction for days to beginning anthesis (Zadoks 60), grain yield and TKW. For grain yield, test weight, FDK, and PKI, there was a significant (*P*<0.05) planting timing by harvest timing interaction. There was a significant (*P*<0.05) harvest timing by cultivar interaction for test weight. Therefore, three reduced models were examined.

The first reduced model examined (PROC GLIMMIX; SAS v9.4) specified the main effect as a fixed effect and year, environment, and replication as random effects for all dependent variables for the in-furrow phosphorus main effect and for spikes m^-2^, spikelets spike^-1^, FHB incidence, FHB severity, FHB index, test weight, FDK, PKI, and DON contamination for the seeding rate main effect, as well as TKW and DON contamination for the harvest timing main effect. The second reduced model (PROC GLIMMIX; SAS v9.4) specified the main effect and main effect by planting timing interaction as a fixed effects and year, environment, and replication as random effects for days to beginning anthesis, grain yield, and TKW for the seeding rate main effect and for grain yield, test weight, FDK, and PKI for the harvest timing main effect. The third reduced model specified the main effect and main effect by cultivar interactions as a fixed effect year, environment, and replication as random effects for test weight for the harvest timing main effect.

Least squares means (LSmeans) were separated with the “lines” option and adjusted with the Tukey–Kramer method. LSmeans of the arcsine FDK ratings were sine transformed to obtain mean FDK ratings; means separation was based on analysis of arcsine transformed data. LSmeans of the log transformed DON, FHB incidence, FHB severity, and FHB index data were raised to the power of 10 to obtain mean DON, FHB incidence, FHB severity, and FHB index; mean separation was based on analysis of log transformed data.

## Results

### In-Furrow Phosphorus

In-furrow phosphorus had an effect on spikes m^-2^ and yield. When 47 kg P_2_O_5_ ha^-1^ was placed in-furrow, spikes m^-2^ increased by 27 spikes m^-2^ when compared to the treatment that lacked in-furrow phosphorus (**Table 3**). The use of in-furrow phosphorus increased yield by 144 kg ha^-1^ compared to the treatment that did not have phosphorus (**Table 4**). Despite the significant (*P*=0.0333) yield increase, wheat began flowering (Zadoks 60) at the same time, about 2 days after heading (**Table 3**). In addition, traits associated with FHB symptomology (FHB incidence, FHB severity, FHB index, FDK rating, DON contamination, and PKI), number of spikelets spike^-1^, TKW, and test weight did not differ regardless of phosphorus treatment (**Tables 3** and **4**).

**Table 3:** Mean of spikes m^-2^, spikelets spike^-1^, Fusarium Head Blight (FHB) incidence, FHB severity, FHB Index, and days to beginning anthesis (Zadoks 60) for soft red winter wheat with differing treatments of in-furrow phosphorus and seeding rate at Princeton, KY, USA, from 2017 to 2019.

Main Effect	Spikes m^-2^	Spikelets spike ^-1^	FHB incidence (%)	FHB Severity (%)	FHB Index	Days to Beginning Anthesis (Zadoks 60)	
**In-Furrow Phosphorus**							
**0 kg ha^-1^**	637 B^a^	13.8	17.2	14.4	3.8	2.4	
**47 kg ha^-1^**	664 A	13.8	17.5	14.0	3.9	2.3	
***P > F***	0.0067	0.8069	0.9025	0.6740	0.950	0.1151	
						Planting Timing^b^	
**Seeding Rate**						October	November
**377 plants m^-2^**	634 B	13.0 A	24.5 A	16.1	5.6	2.0 C	3.0 A
**603 plants m^-2^**	675 A	12.3 B	23.7 B	16.7	5.5	2.0 C	2.5 B
***P > F***	<0.0001	<0.0001	0.0224	0.0750	0.2294	0.0057	
**Cultivar** **^c^**							
**Susceptible**	658	14.1 A	16.3 B	14.9 A	3.8	5.3 B	
**Resistant**	644	13.5 B	18.4 A	13.5 B	3.9	5.9 A	
***P > F***	0.1802	<0.0001	0.0097	0.0050	0.3069	<0.0001	

a means with a different letter within the same column and main effect are significantly different (P<0.05).

b means with a different letter within the planting timing by main effect interaction are significantly different (P<0.05).

c In harvest year 2017 the moderately susceptible cultivar used was Cumberland (Kentucky Small Grain Growers Association, Eastwood, KY), in 2018 and 2019 the moderately susceptible cultivar used was Pioneer 26R53 (Corteva, Johnston IA). The moderately resistant cultivar used all three years was Pembroke 2016 (Kentucky Small Grain Growers Association, Eastwood, KY).

**Table 4:** Means of yield, test weight, thousand kernel weight, Fusarium damaged kernel ratings, percent kernel infection, and deoxynivalenol for soft red winter wheat with different in-furrow phosphorus, seeding rate, harvest timing, and cultivar treatments, at Princeton, Kentucky, USA from 2017 to 2019.

Main Effect	Yield(kg ha^-1^)		Test weight(kg m^-3^)		Thousand Kernel Weight (g)		Fusarium Damaged Kernel Rating (%)		Percent Kernel Infection (%)		Deoxynivalenol (ppm)
**In-Furrow Phosphorus**											
**0 kg ha^-1^**	4780 B^a^		672		30.3		10.8		33		2.1
**47 kg ha^-1^**	4920 A		673		30.3		10.4		33		2.0
***P > F***	0.0333		0.8212		0.7587		0.5073		0.5410		0.5009
	Planting Timing^b^				Planting Timing						
**Seeding Rate**	October	November			October	November					
**377 plants m^-2^**	5120 A	3380 C	671		31.0 B	31.2 A	12.4		24		2.4
**603 plants m^-2^**	5130 A	3650 B	674		30.5 C	31.4 A	11.9		24		2.3
***P > F***	0.0148		0.0761		0.0422		0.1019		0.3945		0.0765
	Planting Timing		Planting Timing				Planting Timing		Planting Timing		
**Harvest timing**	October	November	October	November			October	November	October	November	
**13 to 15% grain moisture**	5450 A	3990 C	694 A	662 C	29.9 B		8.3 B	14.4 A	29 C	37 A	1.7 B
**20 to 22% grain moisture**	5640 A	4280 B	683 B	647 D	30.7 A		5.7 C	14.1 A	30 C	35 B	2.4 A
***P > F***	0.0004		0.0171		<0.0001		0.0034		0.0010		<0.0001
**Cultivar^c^**											
**Susceptible**	4730 B		–		29.1 B		11.8 A		34 A		2.3 A
**Resistant**	4960 A		–		31.5 A		9.4 B		31 B		1.7 B
***P > F***	0.0007		–		<0.0001		<0.0001		<0.0001		<0.0001

a means with a different letter within the same column and main effect are significantly different (P<0.05).

b means with a different letter within the planting timing by main effect interaction are significantly different (P<0.05).

c In harvest year 2017 the moderately susceptible cultivar used was Cumberland (Kentucky Small Grain Growers Association, Eastwood KY), in 2018 and 2019 the moderately susceptible cultivar used was Pioneer 26R53 (Corteva, Johnston IA). The moderately resistant cultivar used all three years was Pembroke 2016 (Kentucky Small Grain Growers Association, Eastwood, KY).

### Seeding Rate

The seeding rate by planting timing interaction indicated that the 603 plants m^-2^ seeding rate planted in November was at beginning anthesis (Zadoks 60) 0.5 days earlier than to the 377 plants m^-2^ seeding rate planted in November (**Table 2**). The October planted wheat did not differ in number days (2.0) to Zadoks 60 for either seeding rate (**Table 3**). There was no difference in days to Zadoks 58 or Zadoks 68 (data not shown).

Seeding rate had a small but significant effect on FHB incidence, in which incidence decreased (*P*=0.0224) by 0.8% for the 603 plants m^-2^ seeding rate compared to the 377 plants m^-2^ seeding rate (**Table 3**). Fusarium head blight severity (*P*=0.075), FHB index (*P*=0.2294), FDK ratings (*P*=0.1019), PKI (*P*=0.3945), and DON contamination (*P*=0.0765) were not affected by seeding rate (**Tables 3** and **4**).

Seeding rate had an effect on the yield components of spikes m^-2^, spikelets spike^-1^ and TKW. When the 603 plants m^-2^ seeding rate was used, spikes m^-2^ increased (*P*<0.0001) by 41 spikes m^-2^ compared to the 377 plants m^-2^ seeding rate (**Table 3**). The 603 plants m^-2^ seeding rate had fewer (*P*<0.0001) spikelets spike^-1^ (12.3) compared to the 377 plants m^-2^ seeding rate (13.0) (**Table 3**). Wheat planted in October had TKW decreased by 0.5 g for the 603 plants m^-2^ seeding rate compared to the 377 plants m^-2^ seeding rate, while the November planted wheat TKW did not differ (*P*=0.0422) for either seeding rate (**Table 4**). For the wheat planted in November, yield increased (*P*=0.0148) by 279 kg ha^-1^ for the 603 plants m^-2^ seeding rate compared to the 377 plants m^-2^ seeding rate. The October planted wheat yield was significantly (*P*=0.0148) greater than the November wheat yields at each seeding rate, however the October 603 plants m^-2^ seeding rate did not differ from the October 377 plants m^-2^ seeding rate (**Table 4**).

### Harvest Timing

Harvest timing affected DON contamination, grain yield and grain quality. Wheat harvested at 20 to 22% grain moisture had increased (*P*<0.0001) DON contamination by 0.7 ppm compared to wheat harvested at 13 to 15% grain moisture (**Table 4**). Grain yield, test weight, FDK ratings, and PKI indicated a harvest timing by planting timing interaction. The November early harvest had an increase in grain yield of 290 kg ha^-1^ compared to the November normal harvest; in contrast, the October early harvest and October normal harvest had the highest yields and did not differ (*P*=0.0004) (**Table 4**). The October normal harvest had the greatest test weight overall (694 kg m^-3^), followed by the October early harvest (683 kg m^-3^), then the November normal harvest (662 kg m^-3^), and lastly the November early harvest (647 kg m^-3^) (**Table 4**). The October early harvest FDK rating was 2.6% less than the October normal harvest rating, however both were significantly (*P*=0.0034) less than the November early harvest (14.1%) and the November normal harvest (14.4%) (**Table 4**). The November normal harvest had the greatest (*P*<0.0001) PKI (37%), followed by the November early harvest (35%), while both the October early harvest (30%) and normal harvest (29%) were significantly less than November early harvest and normal harvest (**Table 4**). Wheat harvested at 20 to 22% grain moisture increased TKW by 0.8g compared to wheat harvested at 13 to 15% grain moisture (**Table 4**). There was a harvest timing by cultivar interaction for test weight. The early harvest susceptible cultivar had the lowest test weight compared to the normal harvest susceptible cultivar, and the resistant cultivar at both harvest timings (*P*=0.0004) (**Table 5**).

**Table 5:** Means of test weight with different harvest treatments at Princeton, Kentucky, USA from 2017 to 2019.

Main Effect	Test weight(kg m^-3^)	
	Cultivar	
**Harvest timing**	Susceptible	Resistant
**13 to 15% grain moisture**	677 A^a^	679 A
**20 to 22% grain moisture**	659 B	674 A
***P > F***	0.0004	

a means with a different letter within the same main effect are significantly different (P < 0.05).

### Cultivar

The main effect of cultivar had an effect on anthesis uniformity, FHB symptomology and grain yield when averaged over all environments and years. The moderately susceptible cultivar began to flower (Zadoks 60) (*P*=<0.0001) 0.6 days earlier than the moderately resistant cultivar (**Table 3**). The moderately resistant cultivar had an increase (*P*=0.0097) in 2.1% FHB incidence, but decreased (*P*=0.0050) FHB severity by 1.4% compared to the moderately susceptible cultivar (**Table 3**). However, both cultivars did not differ (*P*=0.3069) in FHB index. The moderately resistant cultivar reduced (*P*<0.0001) FDK ratings by 24% and reduced (*P*<0.0001) PKI by 3% compared to the moderately susceptible cultivar (**Table 4**). The moderately resistant cultivar decreased (*P*<0.0001) DON contamination by 0.6 ppm compared to the moderately susceptible cultivar (**Table 4**). The moderately resistant cultivar had a significantly (*P*<0.0001) less spikelets spike^-1^ compared to the moderately susceptible cultivar, however both cultivars did not differ (*P*=0.1802) in spikes m^-2^ (**Table 3**). The moderately resistant cultivar had an increased (*P*<0.0001) TKW (31.5 g) compared to the moderately susceptible cultivar (29.1 g) and had an increase (*P*=0.0007) in grain yield of 230 kg ha^-1^ compared to the moderately susceptible cultivar (**Table 4**).

When each cultivar was examined separately for the inoculated and non-inoculated environments similar trends were found, but the magnitude of the differences was greater (data not shown). For FDK the moderately susceptible cultivar was 0.6% greater in the ambient environment and 3.8% greater in the inoculated environment when compared to the moderately resistant cultivar: 4.1 and 15.3% for the moderately resistant cultivar and 4.7 and 19.1% for the moderately susceptible cultivar in the ambient and inoculated environments, respectively. Deoxynivalenol was similar for both cultivars in the ambient environment: 0.5 and 0.6 ppm for the moderately resistant and moderately susceptible cultivars, respectively. However, in the inoculated environment DON was greater for the moderately susceptible cultivar (4.0 ppm) than for the moderately resistant cultivar (3.1 ppm). In addition, test weight and yield of the moderately susceptible cultivar was consistently less than the moderately resistant cultivar in the inoculated environment, while consistent differences were not detected in the ambient environment.

## Discussion

### In-Furrow Phosphorus 

Grain yield and spikes m^-2^ increased when 47 kg ha^-1^ P_2_O_5_ was applied in-furrow at planting. This result was interesting, in that grain yield and spikes m^-2^ increased due to the phosphorus application because all environments of this study had soil-test phosphorus levels that have been identified as adequate to support wheat production in Kentucky (Ritchey and McGrath, 2018). Knapp and Knapp (1978) observed an increase in spike m^-2^ and yield from fall applied phosphorus on adequate phosphorus soils, while Halvorson and Havlin (1992), and Oakes et al. (2016) only observed an increase in yield as rates of phosphorus fertilizer increased in adequate phosphorus soils. However, a majority of yield response due to fall applied phosphorus have occurred in phosphorus deficient soils (Blue et al., 1990; Sander and Eghball, 1999; Karamanos et al., 2003; Chen et al., 2019). In addition to yield increase, Chen et al. (2019) observed an increase in the number of fertile primary tillers when phosphorus was applied in phosphorus deficient soils. There have been anecdotal observations from Canada that in-furrow phosphorus applied at planting as a starter fertilizer have increased wheat heading and anthesis uniformity (Hooker, 2015). In-furrow phosphorous did not increase heading and anthesis uniformity when measured over heading duration, days to beginning anthesis (Zadoks 60), days to full anthesis (Zadoks 68), and total anthesis duration (data not shown), nor did it decrease DON contamination and FHB symptomology (**Tables 3** and **4**). This lack of response is due in part to the reduced response of phosphorus application to winter wheat in soils with adequate phosphorus (Fiedler et al., 1989; Karamanos et al., 2003; Grove et al., 2018).

Starter phosphorus has been observed to improve plant health and yields in stressful situations. For example, in late planted wheat in adequate phosphorus soils, the addition of starter fertilizer with phosphorus increased grain yield in late planted winter wheat (Knapp and Knapp, 1978; Oakes et al., 2016). Similar results are reported in deficient phosphorus soils by Blue et al. (1990) and Sander and Eghball (1999). In-furrow phosphorus did not increase grain yield for the November planted wheat in the current study (data not shown). Blue et al. (1990) also reported that starter phosphorus reduced the negative effects of a low seeding rate. However, there was not a response in grain yield when in-furrow phosphorus was used at the 377 plants m^-2^ seeding rate planted in November (data not shown). The response of phosphorus in stressful growing conditions can be attributed to the wheat plants stimulated root development and early tiller growth from the starter phosphorus (Knapp and Knapp, 1978; Sander and Eghball, 1999; Teng et al., 2013; Oakes et al., 2016).

### Seeding Rate

The increased seeding rate of 603 plants m^-2^ decreased the number of days to beginning anthesis (Zadoks 60). Previous studies have observed an earlier anthesis date with a higher seeding rate (Geleta et al., 2002) as well as a shortened anthesis period (Schaafsma and Tamburic-Ilincic, 2005). Additional studies from Canada have shown that increasing the seeding rate increases main stem numbers and shortening flowering by creating a less variable wheat field at fungicide application (Beres et al., 2018). Increased fungicide coverage could decrease FHB incidence and severity and reduce DON contamination. Although, there was no difference in days to heading, Zadoks 58 (2.5 days), and days to full flower, Zadoks 68, (2.2 days) for the 603 plants m^-2^ seeding rate in the present study.

Although there was no observable difference in days to heading and days to full flower, the more uniformed beginning flowering may have contributed to the decrease in FHB incidence in the 603 plants m^-2^ seeding rate. Schaafsma and Tamburic-Ilincic (2005) observed a shortened anthesis period from an increased seeding rate; however the more uniform anthesis increased FHB index and DON contamination. There was no difference in FHB severity, FHB index, FDK rating, PKI, and DON contamination in the present study, suggesting that the increased uniformity did not increase FHB regardless of disease pressure. The inoculated environments averaged FHB indexes of 5 and 16, with and without fungicides respectively, while both ambient environments each averaged an FHB index of 1 (unpublished data). Even in the high disease pressure situation, (inoculated, no fungicide application), DON contamination did not differ for seeding rate (data not shown). The decreased FHB incidence and the lack of difference for DON contamination and other FHB symptomology measurements indicates that there is potential for a more uniform anthesis without increasing FHB symptomology.

In addition to altering heading and anthesis uniformity, an increased seeding rate can affect yield and yield components. Yield is determined by the number of spikes per unit area, the number of kernels per spike, and the weight of those kernels (Kiesselbach and Sprague, 1926). Increasing the seeding rate to 603 plants m^-2^ increased grain yield especially in the November planted wheat, and altered the yield components by increasing the number of spike m^-2^, decreasing the number of spikelets spike^-1^ and decreasing the TKW for October planted wheat. Similar results have been reported where a higher seeding rate increased grain yield and spikes m^-2^ (Blue et al., 1990; Geleta et al., 2002; Lloveras et al., 2004; Otteson et al., 2008). However, there have been mixed findings on spikelets spike^-1^ and TKW response to seeding rate. Some studies observed an increase in kernels spike^-1^ and increased kernel weight (Blue et al., 1990; Geleta et al., 2002), while others observed a decrease in kernels per spike and decreased kernel weight (Lloveras et al., 2004; Ma et al., 2018). Although spikelets spike^-1^ and TKW decreased, increasing the seeding rate increases the number of spikes m^-2^, increasing the proportion of yield coming from the main stem and primary tiller, as secondary and tertiary tillers decrease (Otteson et al., 2008). Having more main stems and primary tillers while increasing yield, may promote a more uniform heading and anthesis period for better fungicide application, without compromising yield.

Increasing the seeding rate when wheat is grown in stressful environments has improved yields. There was an increase of 269 kg ha^-1^ from the 603 plants m^-2^ seeding rate compared to the 377 plants m^-2^ seeding rate in the November planted wheat. The November planted wheat was exposed to colder temperatures during seed germination and seedling emergence, producing very little vegetation throughout the winter, while the October planted wheat was able to produce adequate tiller growth before winter. This is similar to findings of Oakes et al. (2016) where the on-time planted winter wheat had more fall tiller growth and development, than late planted winter wheat which had more spring tiller growth. Increasing the seeding rate for late planted wheat would increase the number of tillers and spikes produced in the spring, alleviating the negative effects of late planting. This can lead to increased grain yields in late planted winter wheat, although yields were still lower than on-time planted wheat (Blue et al., 1990; Ma et al., 2018).

### Harvest Timing

Harvesting at a targeted 20 to 22% grain moisture decreased FDK ratings in the October planted wheat, and decreased PKI in the November planted wheat. Fusarium damaged kernel ratings and PKI have been shown to increase with the presence of post anthesis moisture, especially 45 days or more after anthesis (Xue et al., 2004; Cowger and Arellano, 2013). There was approximately 100 to 160 mm of precipitation (http://www.kymesonet.org) that occurred between the early harvest timing and normal harvest timing each year. Harvesting 12 to 21 days earlier at high moisture, may have prevented the additional development of FDK and PKI by removing the grain from the FHB conducive environment. These findings are similar to anecdotal observations by Kentucky wheat producers where harvesting at greater than 15% grain moisture has led to greater grain quality. 

Harvesting at a targeted 20 to 22% grain moisture decreased FDK and PKI in certain situations, but DON contamination did not decrease compared to harvesting at 13 to 15% grain moisture. This was somewhat unexpected as FDK is strongly associated with DON contamination (Paul et al., 2005) and there was a decrease in FDK ratings in the October early harvest. The increase in DON contamination at the higher grain moisture does not agree with some Kentucky wheat growers’ anecdotal observations. However there have also been reports by Cowger and Arellano (2013) and Culler et al. (2007) of DON contamination declining during grain fill and harvest maturity. Deoxynivalenol is thought to be a virulence factor in *F. graminearum* infection and that DON biosynthesis occurs shortly after infection (Hallen-Adams et al., 2011). Deoxynivalenol biosynthesis gene expression was greatest directly after infection and during fungal colonization, but diminished as the plants matured (Hallen-Adams et al., 2011). It is proposed that DON is removed from the host by a detoxification method (Audenaert et al., 2013) or through leaching *via* free water movement from the plant (Culler et al., 2007; Gautam and Dill-Macky, 2012). These proposed methods could be possible in the current study as precipitation events occurred between the early and normal wheat harvest. Another possibility of increased DON in the high moisture grain, is that less tombstone kernels were blown out during combine harvest. Thousand kernel weights were increased for grain harvested at 20 to 22% grain moisture compared to grain harvested at 13 to 15% grain moisture. Combine settings can be adjusted to increase fan speed to increase the potential of tombstone kernels blown out of the combine, thus increasing the quality of the grain in the tank (Salgado et al., 2011). The heavier tombstone kernels at early harvest may not have been light enough to be blown out of the combine, potentially leading to the increased DON contamination.

Harvesting at a targeted 20 to 22% grain moisture increased grain yield in the November planted wheat and increased TKW overall. The October planted wheat yield was not effected by harvesting at 20 to 22% grain moisture. Test weights were decreased at each planting timing when harvested at 20 to 22% grain moisture (**Table 4**). This is expected as test weight is somewhat inversely proportional to grain moisture content; as grain moisture decreases test weight will increase until the maximum test weight is reached at approximately 14 to 15% grain moisture (Nelson, 1980; Brooker et al., 1992). When grain is harvested at elevated grain moisture content, drying the grain to 13 to 15% grain moisture can increase the final test weight (Brooker et al., 1992). It is important to note that wheat grain harvested at the targeted 20 to 22% grain moisture must be dried soon after harvest to prevent spoilage and mycotoxin accumulation as *F. graminearum* can continue to grow and produce DON in harvested grain at grain moistures above 17% (Hope et al., 2005; McNeill et al., 2009).

Test weight also decreased in the susceptible cultivar when it was harvested at 20 to 22% grain moisture however; there was no difference in test weights of the resistant cultivar (**Table 5**). The decrease in test weight of the susceptible cultivar at high grain moisture was unexpected. The grain moisture may have influenced this decrease; however, the resistant cultivar did not have a decrease in test weight when harvested early. It possibly could be influenced by poor quality grain, as this is the FHB susceptible cultivar. Salgado et al. (2015) reported test weight decreased at least 5% at an FHB index level of 10. The Kentucky environments averaged a FHB index level of 6, which may have influenced the 2.6% reduction in test weight of the high moisture test weight of the susceptible cultivar. Salgado et al. (2015) also observed the test weight of the resistant cultivar test weight was less influenced by FHB severity and FHB index compared to the test weights of the susceptible cultivar. The same trends were observed in the high moisture harvest between the susceptible and resistant cultivars. With these trends, winter wheat producers should use a FHB-resistant cultivar to help preserve test weight.

Harvesting at 20 to 22% grain moisture of on-time planted wheat did not decrease grain yield, and only reduced test weight by 1.6%. The October early harvest had increased TKW, reduced FDK, had no difference in PKI, and greater DON contamination compared to the October normal harvest. When wheat producers sell grain, dockage can occur when standards are not met for grain moisture, test weight, and DON contamination. If producers have a grain drying system on farm, they can dry their grain to 12.5% and increase their test weight, creating less dockage when selling grain; however, they would not be able to change the DON contamination in the grain. Harvesting at 20 to 22% grain moisture provides the potential to plant double crop soybeans after wheat harvest, 12 to 21 days earlier, where the increase in soybean yield could potentially pay for the drying cost and dockage and still retain a profit. 

### Cultivar

The moderately resistant cultivar, Pembroke 2016, was used in all three years, whereas the moderately susceptible cultivar changed between the years (Cumberland in 2017, and Pioneer 26R53 in 2018 and 2019). Cumberland and Pioneer 26R53 had similar high FHB disease ratings on a 1 to 9 scale where 1 is resistant and 9 is susceptible from the University of Kentucky Variety Test, for the years they were respectively evaluated (Bruening et al., 2009; Bruening et al., 2017). Cultivar resistant type had a large effect on many of the parameters measured in this study. In the present study the moderately resistant Pembroke 2016 had significantly lower FHB severity, FDK rating, PKI, and DON contamination compared to the susceptible cultivar. These findings are similar to the findings of the 2017 Kentucky Wheat Variety Test where Pembroke 2016 had a more resistant score for FHB compared to Pioneer 26R53 (Bruening et al., 2017) and support the findings that genetic resistance greatly influences the extent of FHB symptomology observed among different cultivars (Willyerd et al. (2012) and Wegulo et al (2011).

Reduced FHB symptomology in wheat plants can affect grain yield. Wegulo et al. (2011) observed a negative correlation between yield and FDK, yield and FHB index, and yield and DON, respectively. In the present study the moderately resistant cultivar had reduced FDK, PKI, DON and FHB severity compared to the moderately susceptible cultivar, which lead to an increase in grain yield, test weight, and TKW. Similar results were observed when data analyses were conducted over the environment type (artificially infested with *F. graminearum* inoculum or without artificially infestation). The moderately resistant cultivar had reduced FHB symptomology compared to the moderately susceptible cultivar which led to an increase in grain yield and grain qualities when severe FHB pressure occurs (data not shown). Thus, the use of moderately resistant cultivars continues to be a one of the most effective management practices to control FHB and DON contamination, and increase grain yield and quality, in winter wheat.

## Conclusions

This study investigated integrating additional management practices of in-furrow phosphorus, seeding rate, and harvest timing to decrease DON contamination in the grain. Our results indicated that in-furrow phosphorus did not affect DON contamination but did increase grain yield and spikes m^-2^. The 603 plants m^-2^ seeding rate decreased the number of days to beginning anthesis in the November planted wheat and decreased FHB incidence, however there was no difference in DON contamination, PKI, or FDK ratings. The 603 plants m^-2^ seeding rate did increase yields in the November planting timing, indicating that in late planted wheat increasing the seeding rate will decrease the negative impacts of late planting. Overall, application of in-furrow phosphorus and increased seeding rate had little effect on heading and anthesis uniformity. Harvesting at 20 to 22% grain moisture did not affect the October planted grain yield, while it did preserve the November early harvest grain yield. Test weight decreased with the high moisture harvest, however TKW increased while FDK and PKI decreased in certain situations compared to the normal moisture harvest. Harvesting at 20 to 22% grain moisture led to greater DON contamination than harvesting at 13 to 15% grain moisture. 

Although application of in-furrow phosphorus and increased seeding rate did not affect DON contamination and harvesting at 20 to 22% grain moisture resulted in greater DON contamination, these treatments increased grain yield and decreased days to anthesis and PKI in more stressful environments. Thus, there is potential for these treatments to be used to reduce the negative effects of planting wheat late.

## Data Availability Statement

The raw data supporting the conclusions of this article will be made available by the authors, without undue reservation.

## Author Contributions

KR, CB, DS, and CK conceived the research project and experimental design. KR and CK conducted the field and laboratory experiments. CB and DS provided guidance and field and laboratory space and equipment. KR analyzed the data, interpreted the results, and wrote the manuscript under the guidance of CK and with contributions from CB and DS.

## Funding

Funding for this project was provided by the Kentucky Small Grain Growers Promotion Council and was greatly appreciated.

## Conflict of Interest

The authors declare that the research was conducted in the absence of any commercial or financial relationships that could be construed as a potential conflict of interest.
